# Programmed death ligand-1 (PD-L1) expression in meningioma; prognostic significance and its association with hypoxia and NFKB2 expression

**DOI:** 10.1038/s41598-020-70514-z

**Published:** 2020-08-24

**Authors:** Shirin Karimi, Sheila Mansouri, Yasin Mamatjan, Jeff Liu, Farshad Nassiri, Suganth Suppiah, Olivia Singh, Kenneth Aldape, Gelareh Zadeh

**Affiliations:** 1 Princess Margaret Cancer Center, MacFeeters-Hamilton Center for Neuro-Oncology Research, 14-701, Toronto Medical Discovery Tower (TMDT), 101 College St, Toronto, ON M5G 1L7 Canada; 2 National Cancer Institute, Building 10, Room 2S235, Bethesda, MD 20892-1500 USA

**Keywords:** Molecular medicine, Oncology

## Abstract

Management of clinically aggressive meningiomas is a considerable challenge. PD-L1 induced immune suppression has increasingly gained attention in clinical management of cancer; however, to date, the clinical significance and regulatory mechanisms of PD-L1 in meningioma is not yet fully characterized. We sought to characterize PD-L1 expression in meningioma and elucidate its regulatory mechanisms. Immunohistochemical staining of PD-L1 expression in meningiomas showed 43% positivity in both tumor and immune cells and we observed intra and inter tumoral heterogeneity. Univariate and multivariate analyses confirmed that PD-L1 protein expression is an independent prognostic marker for worse recurrence free survival in meningioma. Furthermore, our transcriptomic analysis revealed a strong association between PD-L1 expression and that of NFKB2 and carbonic anhydrase 9 (CA9). We also demonstrated that both of these markers, when co-expressed with PD-L1, predict tumor progression. Our studies on several meningioma cell lines cultured in hypoxic conditions validated the association of CA9 and PD-L1 expression. Here we show the clinical significance of PD-L1 in meningioma as a marker that can predict tumor recurrence. We also show an association PD-L1 expression with NFKB2 expression and its induction under hypoxic conditions. These findings may open new avenues of molecular investigation in pathogenesis of meningioma.

## Introduction

Meningiomas represent the most common primary intracranial brain tumor in adults and comprise 37% of all central nervous system tumors^1^. The World Health Organization (WHO) classifies meningiomas into three histologic groups including benign (grade I), atypical meningiomas (WHO grade II) and malignant meningioma (WHO grade III), which the last two groups represent almost 20% of all meningioma cases. The two latter groups typically show an aggressive clinical course, especially with high recurrence rates^2,3^. Prediction of tumor recurrence and management of the clinically aggressive behaviour in meningiomas remains a major challenge in neuro-oncology practice. Several studies have demonstrated that meningiomas—even those that are histologically benign—are a heterogeneous group of tumors that harbour various somatic mutations^4,5^ and different clinically significant methylation signatures^6,7^. Routine implementation of the methodology to detect these alterations in clinical setting, however, requires broader adoption. Therefore, identifying key markers that can be utilized in routine practice to predict outcome and offer new avenues for treatment of aggressive meningiomas is urgently needed.

It has been shown that tumor progression is a result of a balance between tumor growth and tumor-induced immune suppression, with one of the main mechanisms for tumor-induced immune suppression being activation of the PD-1/PD-L1 checkpoints. Currently, PD1/PD-L1 immune checkpoint blockage therapy has dramatically improved the overall survival of patients in specific cancer types and has been emerged as a standard of care for specific cancer types. Interaction of PD-1 and PD-L1 on tumor cells, lymphocytes, dendritic cells, and macrophages causes immune tolerance and tumor escape^8,9^. Several mechanisms can potentially induce PD-L1 expression including hypoxia, activation of oncogenic and inflammatory signalling pathways, release of cytokines, and epigenetic regulatory mechanisms^8,10–12^. Notably, PD-L1 expression has not only been utilized to predict response to immune checkpoint therapy but it also displays prognostic significance for tumor progression in several cancers^8,13,14^.

Several recent investigations in meningioma using immunohistochemical staining suggest a role for PD-L1 expression in immune suppression within the tumor microenvironment in high-grade meningioma. These studies in meningiomas have utilized tissue microarrays to demonstrate PD-L1 expression in meningioma and uncover its role in immune suppression within the tumor microenvironment in high-grade meningioma^15–18^. Furthermore, Dunn et al. reported a patient with a recurrent meningioma WHO grade II with a dramatic response to checkpoint inhibitor therapy^19^. Therefore, there is a potential for clinical utility of PD-L1 IHC expression in clinical management of meningioma patients.

It has been shown that PD-L1 expression using different antibodies and in small tissue samples might not be representative of the entire tumor specimen because of tumor heterogeneity. In this regard, detailed assessment of percentage of positivity and pattern of PD-L1 expression in clinical resection specimens in meningioma are still lacking^20–22^.

Here, we focused on clinical and pathological characterization of PD-L1 expression on whole sections of meningioma cases and correlated PD-L1 expression with patient outcome to gain a better understanding of its prognostic clinical utility and identify its potential regulatory mechanisms in meningiomas.

## Result

### Patient characteristics

We selected a total of 93 meningioma patients for this study, including 74 primary and 19 recurrent tumors with the available tumor tissue from the department of pathology; University Health Network. The female to male ratio was 1.6 (58/35) and median age at the time of diagnosis was 59 years (range 25–87). The histopathologic review of the cohort was conducted by two independent neuropathologists (SK and KA) using the 2016 WHO classification scheme for meningioma. The cohort was enriched with high grade meningiomas; including 44% (41/93) WHO grade I, 46% (43/93) WHO grade II, and 10% (9/93) WHO grade III cases. The maximum tumor diameter (MTD) ranges from 1.1 to 8.1 cm with the median of 4.6 cm. The follow up clinical data to assess the tumor recurrence were available for 88 cases and 48% (42/88) of patients had tumor recurrence based on serial post-op MRIs and clinical notes. The details of the clinic-pathologic features are shown in Table 1. Based on univariate analyses, we demonstrated that consistent with previous reports, WHO grading, extent of resection, plus maximum tumor diameter (MTD) were prognostic for tumor recurrence in our cohort (Table 1).

**Table 1:** Cohort characteristics.

Character	N (%)	Median/range	Univariate analysis			Multivariate analysis*		
			*p*	HR	CI (95%)	*p*	HR	CI (95%)
Age (years)		59 (25–87)	0.6	1.02	0.9–1			
Gender (Female)	58 (62)							
**Tumor location**			0.4	0.73	0.31–1.72			
Convexity	43 (47)							
Skull based	14 (15)							
Falcine and parasagital	10 (11)							
Other	25 (27)							
**WHO grade**			0.005	1.85	1.20–2.85	0.04	1.76	1.01–3.04
WHO I	41 (44)							
WHO II	43 (46)							
WHO III	9 (10)							
**Extent of resection**			0.045	1.9	1.01–3.56	0.01	2.7	1.23–5.97
Gross total resection	54 (58)							
Subtotal resection	39 (42)							
Maximum tumor diameter (MTD) cm		4.6 (1.1–8.1)	0.02	1.28	1.03–1.59	0.26	1.13	0.9–1.41
Brain invasion	19 (20%)		0.2	1.61	0.77–3.34			
Primary (newly diagnosed tumor)	74 (80)		0.21	0.65	0.33–1.28			
Prior radiotherapy	21 (22)		0.29	1.41	0.74–2.70			
Tumor recurrence/progression	42 (48)							
Duration of follow up since surgery (yrs.)		4.43 (0.2–34)						
PD-L1 IHC expression								
Visual scoring (positive/negative)	40 (43%)		< 0.0001	5.14	2.54–10.41	< 0.001	4.9	2.05–11.88
HALO digital scoring (% positive cells)		0.08 (0.00–19.13)	0.012	1.1	1.02–1.19			

*Multivariate analysis of PD-L1 expression.

### Characterization of PD-L1 expression in meningioma

**Figure 1:**
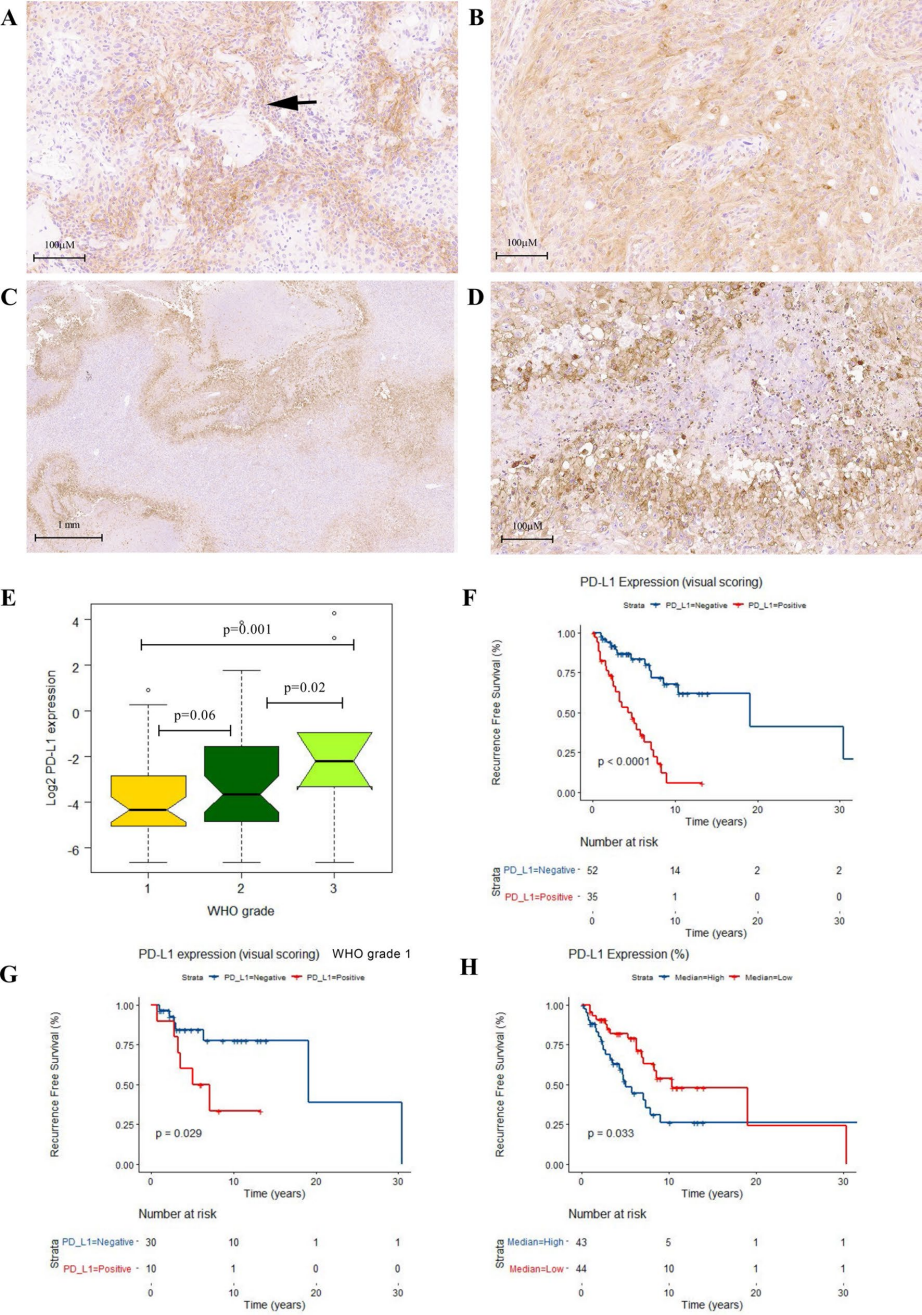
PD-L1 expression in meningioma. Representative images from heterogeneous PD-L1 IHC staining in WHO grade II with both tumor and lymphocytic positive membranous and cytoplasmic reactivity in perivascular areas (black arrow) with intratumoral extension in a WHO grade II (**A**,**B**), significant regional , per necrotic accentuation and intratumoral extension in a malignant meningioma (WHO GIII) (**C**,**D**). **E** Box plot graph (Two way ANOVA test demonstrates significant difference in means of PD-L1 expression (% positive cells) using HALO digital analysis in WHO grades. Kaplan Meier’s (KM) RFS analysis of PD-L1 expression (Visual scoring) in the meningioma cohort (**F**) and in the meningioma grade I patients (**G**). KM curve for digital HALO analysis of PD-L1 expression in the meningioma cohort (**H**) based on separation into two distinct risk groups high and low by median (0.08%). (R v3.3.1).

In order to evaluate PD-L1 expression in meningiomas, we performed IHC analysis on whole sections from the 93 cases, followed by visual scoring and digital quantification using HALO software (AOMF, UHN, Canada). We observed a heterogeneous intra-tumoral immune-reactivity for PD-L1 in the whole tumor, in both tumor and immune cells (Fig. 1A–D). Both membranous and cytoplasmic positivity was detected upon visual microscopic review. The staining pattern in these meningioma cases showed regional positivity with predominantly consistent perivascular staining specially in WHO grades II and III. Most notably, there was accentuation of immune reactivity in peri-necrotic areas with intra-tumoral extension in atypical and malignant meningiomas. We also observed strong PD-L1 positivity in junctional regions between tumor and dura.

PD-L1 positivity in the whole tumor was detected in 43% (40/93) of our cases upon visual microscopic review, including 27% (11/41) of WHO grade I, 47% (23/43) of WHO grade II, and 67% (6/9) of WHO grade III tumors. Notably, we found a statistically significant difference in distribution of PD-L1 positivity and WHO grade (Chi-squared test, *p* = 0.01). We further performed digital assessment of PD-L1 expression in the same samples and found a median of 0.08% PD-L1 positivity (number of PD-L1 positive cells/total positive and negative cells × 100) across all meningioma cases (range 0.00–19.13%). Based on this median, we found that 52% (48/93) of our cases displayed high PD-L1 positivity. The median PD-L1 positivity was 0.05% in meningioma WHO Grade I versus 9.6% in combined Grades II and III cases. Similar to visual scoring, we detected a statistically significant difference in mean PD-L1 expression (%) in three WHO grades (Two way ANOVA test, F-ratio = 5.5, *p* = 0.005) (Fig. 1E). The t-test between the different grades confirmed the statistically significant difference of PD-L1 expression (%) between benign and malignant (t-test, *p* = 0.001) and also between atypical and malignant meningiomas (t-test, *p* = 0.02). The t-test did not show any significant difference between grade I and II (*p* = 0.06). With respect to PD-L1 positivity, we found over all similar results with both independent visual scoring and digital HALO image analysis of our cases (One way ANOVA test, F-ratio = 7.3, *p* = 0.008).

Additionally, we found that PD-L1 positivity based on digital assessment correlated significantly with patients’ age at the time of surgery (Spearman correlation, R = 0.3, *p* = 0.01) and higher MTD (Spearman correlation, R = 0.3, *p* = 0.006). Notably, the expression of PD-L1 positivity between primary and recurrent meningioma was not significantly different (Chi-squared test, *p* = 0.3). We did not find a significant association between PD-L1 positivity and gender or tumor location.

### Prognostic significance of PD-L1 expression in meningioma

To further evaluate the prognostic significance of PD-L1 positivity in meningioma, we performed Kaplan Meier (KM) survival analysis in meningioma patients dichotomized based on PD-L1 positivity as per visual scoring. Our results indicated that PD-L1 positivity correlated significantly with worse recurrence free survival (RFS) in our cohort (*p* < 0.0001) (Fig. 1F). Interestingly, this parameter using KM univariate analysis was predictive for tumor progression in WHO grade I meningiomas (*p* = 0.03) (Fig. 1G). We then used the median cut-off of 0.08% for PD-L1 expression based on digital analysis and dichotomized the cohort into low (45/93) and high (48/93) for KM survival analysis. Consistent with our visual scoring approach, median PD-L1 positivity of 0.08% served as a prognostic cut-off for prediction for higher risk for tumor recurrence (RFS) (*p* = 0.033; Fig. 1H). Additionally, multivariate analysis for PD-L1 positivity based on visual scoring adjusted for WHO grade, MTD, and extent of resection (EOR) confirmed the independent prognostic significance of this parameter in our cohort (*p* =  < 0.001, HR = 4.9, CI(95%) = 2.05–11.88) (Table 1).

### Cellular pathways associated with PD-L1 expression in meningioma

In order to identify genes and cellular pathways associated with PD-L1 expression in meningiomas, we utilized publically available microarray datasets from 2 GEO studies (Affymetrix Human Genome U133 plus 2.0 Array [HG-U133_Plus_2]): GSE16581 and GSE9438^23,24^. A total of 97 samples were identified, which were dichotomized into high and low PD-L1 expression groups based on the median of normalized PD-L1 mRNA expression. A total of 90 differentially expressed genes were identified among high versus low PD-L1 expressing samples (*p* < 0.05, fold change > 1.5). Subsequent pathway and gene ontology analysis demonstrated that PD-L1 expression is associated with several key cellular pathways listed in Table 2. Specifically, we found that NFKB and hypoxia were among the top cellular pathways associated with higher PD-L1 expression. We then examined the list of genes within each pathway and identified NFKB2, RELB and STAT3, among top candidate genes that positively correlated with high PD-L1 expression. Similarly, the second most notable correlation was found between CA9 and VEGFA mRNA levels, both of which are hallmarks of hypoxic response (Table 3). All of these findings led us to investigate the association of hypoxia, in addition to RELB, pSTAT3 and NFKB2 expressions with PD-L1 expression in our meningioma cohort.

**Table 2:** Pathways associated with PD-L1 mRNA expression (2 GEO studies*).

Description	# Genes in overlap (k)	*p*	FDR q-value
Genes regulated by NFKB in response to TNF	35	5.14e−41	2.57e−39
Genes up-regulated in response to IFNG	32	3.08e−36	7.7e−35
Genes up-regulated during transplant rejection	23	6.51e−23	1.08e−21
Genes defining inflammatory response	20	7.42e−19	9.27e−18
Genes up-regulated in response to alpha interferon proteins	12	6.06e−13	6.06e−12
Genes mediating programmed cell death (apoptosis) by activation of caspases	14	9.73e−13	8.11e−12
Genes encoding components of the complement system, which is part of the innate immune system	15	1.29e−12	9.21e−12
Genes up-regulated by IL6 via STAT3	11	4.43e−12	2.77e−11
Genes defining epithelial-mesenchymal transition, as in wound healing, fibrosis and metastasis	14	1.86e−11	8.45e−11
Genes up-regulated in response to low oxygen levels (hypoxia)	14	1.86e−11	8.45e−11

*Refs.^23,24^.

**Table 3:** Correlation of mRNA expression with PD-L1 in two GEO meningioma data sets (n = 97)*

Gene	Spearman's correlation	*p* (correlation)
PDCD1	0.367321	0.000214712
RELB	0.361825	0.000270987
NFKB2	0.361772	0.000271599
CTLA4	0.342494	0.000594918
CA9	0.329906	0.000966941
VEGFA	0.298864	0.00294341
STAT3	0.236627	0.01962
PDCD1LG2	0.194837	0.0558222
B cells memory	0.310038	0.00199844
Eosinophils	0.29593	0.00325042
NK cells activated	− 0.270964	0.00726367
T cells follicular helper	− 0.191971	0.0595993
T cells CD8	− 0.189315	0.0632835
Dendritic cells resting	− 0.187648	0.065688

*Refs.^23,24^.

### Validation of cellular pathways associated with PD-L1 expression

**Figure 2:**
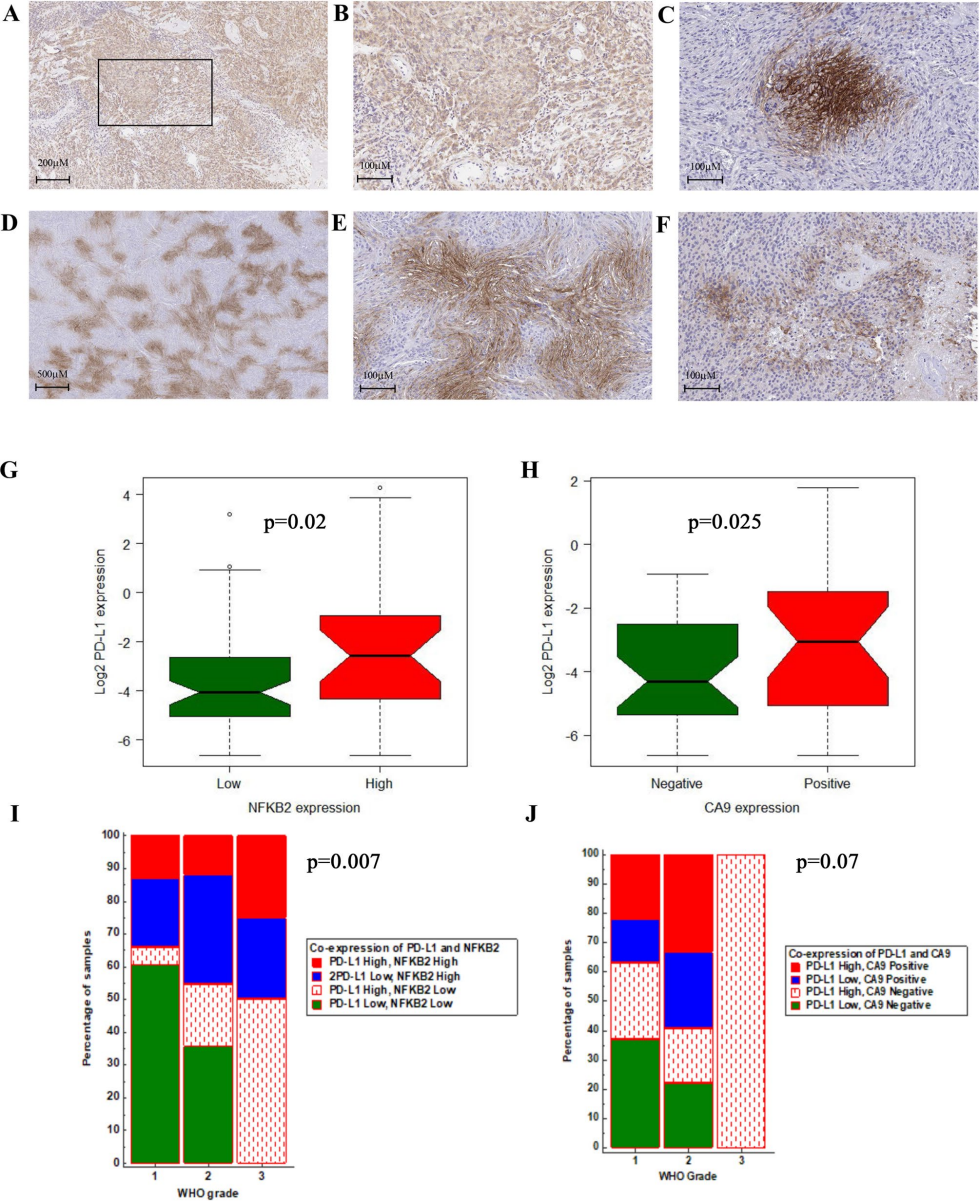
Representative images (× 20) from IHC staining of high cytoplasmic expression of NFKB2 (**A**,**B**) in a meningioma grade II. The CA9 IHC staining shows presence of regional, membranous and cytoplasmic in the tumor; distant from vascular channels in WHO grade II meningioma (**C**,**D**) and also in per necrotic areas of WHO grade III (**E**,**F**). The Box plot shows significant higher percentage of PD-L1 positive cells in meningioma with high NFKB2 expression (**G**). The same analysis in primary meningiomas indicates higher percentage of PD-L1 in positive CA9 meningioma cases (**H**). Chi-squared analysis demonstrated the positive association of WHO grade and co-expression of NFKB2 and PD-L1 in the meningioma cohort (**I**). The same analysis for WHO grade and co-expression of CA9 and PD-L1 (**J**). (R v3.3.1, MedCalc).

We then evaluated the expression of specific candidate proteins: NFKB2, pSTAT3, RELB, and CA9 by immunohistochemistry based on availability of the unstained slides and we correlated their expression with that of PD-L1 in our meningioma cohort. We observed high expression of NFKB2, RELB, pSTAT3, and positive CA9 expression in 30% (26/88), 26% (24/91), 63% (46/73), and 51% (37/72) of the meningioma cases, respectively (Table 4). Of note, we detected cytoplasmic NFKB2 immunoreactivity in all grades of meningioma with a more diffuse pattern in high-grade cases (Grades II and III) (Fig. 2A–B). CA9 displayed both membranous and cytoplasmic expression and highlighted the hypoxic areas in meningioma; patchy in tumor cells that were not directly adjacent to any large vascular channels and also in viable cells around the necrotic areas in anaplastic meningiomas (Fig. 2C–F).

**Table 4:** Characteristics of RELB, NFKB2, pSTAT3 and CA9 IHC expression in the meningioma cohort.

Gene	Number (%)	Univariate analysis for RFS (Cox regression)			Multivariate analysis for RFS*			Association with PD-L1 expression
		*p*	HR	CI (95%)	*p*	HR	CI (95%)	ANOVA test (*p*)
**RELB**	91 (100)	0.14	1.69	0.83–3.43				0.8
Low	67 (74)		1.27	0.79–2.04				
High	24 (26)							
**NFKB2**	88 (100)	0.0004	3.14	1.60–6.14	0.06	2.05	0.96–4.39	0.02
Low	62 (70)							
High	26 (30)							
WHO grade					0.04	1.85	1.03–3.30	
Extent of resection					0.01	2.67	1.24–5.75	
**pSTAT3**	73 (100)	0.13	1.81	0.83–3.97				0.14
Low	27 (37)							
High	46 (63)							
**CA9**	72 (100)	0.02	2.4	1.13–5.09	0.03	2.39	1.09–5.24	0.02**
Negative	35 (49%)							
Positive	37 (51%)							
WHO grade					0.07	1.72	0.95–3.14	
Extent of resection					0.01	2.74	1.23–6.13	

*Multivariate analysis for WHO grade, extent of resection (GTR/STR).

**Primary meningioma.

We then assessed the expression of PD-L1 (% of positive cells) with RELB, NFKB2, pSTAT3 and CA9. The results showed that the patients with high expression of NFKB2 have higher mean of PD-L1 expression in our cohort (One way ANOVA test, F-ratio = 5.1, *p* = 0.02) (Fig. 2G) (Table 4). Although our analysis did not show association of CA9 and PD-L1 in the meningioma cohort (One way ANOVA test, F-ratio = 2.9, *p* = 0.09), we found 46% (26/57) positive CA9 expression in primary meningioma with significant positive relationship with high PD-L1 expression (One way ANOVA test, F-ratio = 5.3, *p* = 0.025) (Fig. 2H). Our analysis in this cohort showed no significant difference in mean of PD-L1 positivity between samples with high or low RELB or pSTAT3 expression (Table 4).

**Figure 3:**
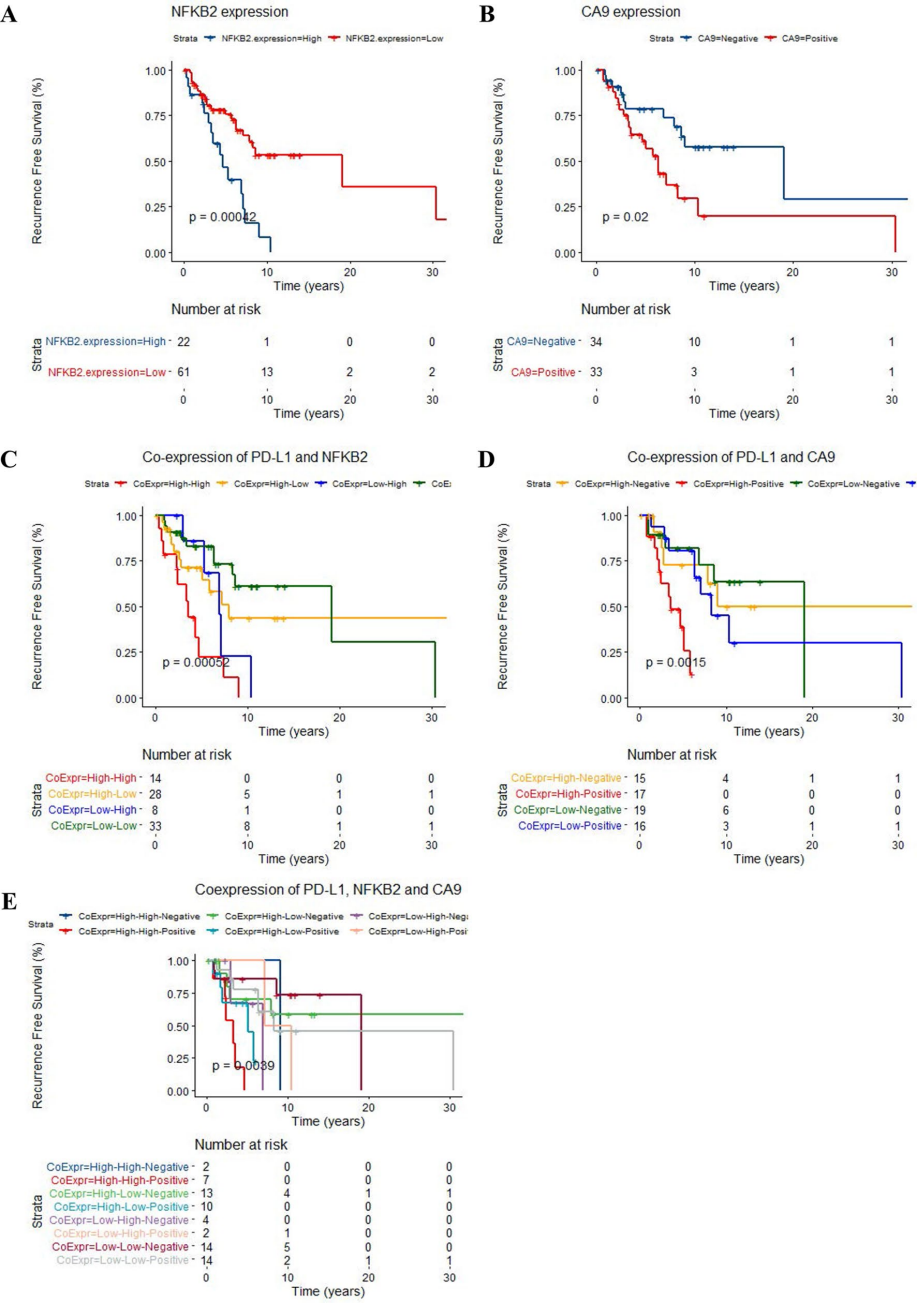
KM curves confirmed the prognostic significance for high NFKB2 expression and CA9 positivity in meningioma cases to predict worse RFS (**A**,**B**). KM analysis of predictive significance expression of these two proteins with PD-L1 in the meningioma cohort (**C**,**D**). **E** KM survival analysis of the IHC expressions of PD-L1, NFKB2 and CA9 in the meningioma cohort: High expression of PD-L1, NFKB2 combined with positive immunoreactivity for CA9 is associated with increased risk for tumor recurrence. (R v3.3.1).

We observed high expression of NFKB2 and CA9 positivity significantly predicted the tumor recurrence in our cohort; *p* = 0.0004 and p = 0.02, respectably (Fig. 3A,B) (Table 4). The univariate survival analyses pSTAT3 and RELB did not show any impact on outcome in our cohort. (Table 4) .Multivariate Cox regression survival analysis adjusted for WHO grade and extent of resection confirmed the independent prognostic significance of CA9 positivity ((*p* = 0.02, HR = 2.4, CI (95%) = 1.13–5.09) and border line significance for NFKB2 expression (*p* = 0.06, HR = 2.05, CI (95%) = 0.96–4.39) in our cases (Table 4).

### Prognostic significance of PD-L1 co-expression with NFKB2 and CA9

We next investigated the clinical and biologic significance of PD-L1 co-expression with NFKB2 or CA9. We classified the cohort into 4 groups based the expression of NFKB2 (low/high) and PD-L1 (%) high and low (median = 0.08%) (Supplemental Table 1). Chi-squared analysis revealed significant difference between co-expression of NFKB2 and PD-L1 in three WHO grades (*p* = 0.007, Fig. 2I).

Similarly, we also categorized the meningioma cases in to 4 subgroups based on CA9 expression (positive/negative) and PD-L1 expression into high and low (median = 0.08%) (Supplemental Table 1). The Chi-squared test did not show any statistically significant difference of co-expression of CA9 and PD-L1 in three meningioma WHO grades (*p* = 0.07, Fig. 2J).

We then used Kaplan Meier survival analysis of patients’ RFS and found that PD-L1 co-expression with NFKB2 (*p* < 0.001) (Fig. 3C) and also with CA9 expression; a hypoxic biomarker significantly predicted tumor progression in our cohort (*p* = 0.001, Fig. 3D) (Table 4).

Our multivariate survival analyses adjusted for WHO grade and extent of resection, indicated the independent prognostic significance of high PD-L1 and NFKB2 co-expression to predict tumor progression (*p* = 0.002, HR = 1.57,CI(95%) = 1.117–2.11) (Supplemental Table 2). Regarding the clinical impact of hypoxia and immunosuppressive effect of PD-L1, the multivariate analysis of co-expression of CA9 and PD-L1 , adjusted for WHO grade and extent of resection confirmed the independent prognostic significance of this parameter; *p* = 0.009, HR = 1.7, CI (0.95%) = 1.14–2.55 (Supplemental Table 2).

We then categorized the meningiomas cases based on co-expression of all three markers: PD-L1 (high/low, median = 0.08%), NFKB2 (high/low), and CA9 (positive/negative) into 8 subgroups (Fig. 3E). Kaplan Meier survival analysis demonstrated higher likelihood for tumor recurrence in meningioma cases co-expressing all three proteins *p* = 0.004. Furthermore, the multivariate analysis adjusted for confounding factors; extent of resection and WHO grade showed that expression of PD-L1, NFKB2 and CA9 is a prognostic factor for worse recurrence free survival in meningioma patients , independent of WHO grade and extent of resection (ANOVA *p* = 0.024) (Supplemental Table 3).

### Induction of PD-L1 expression in meningioma cells lines grown in hypoxic conditions

**Figure 4:**
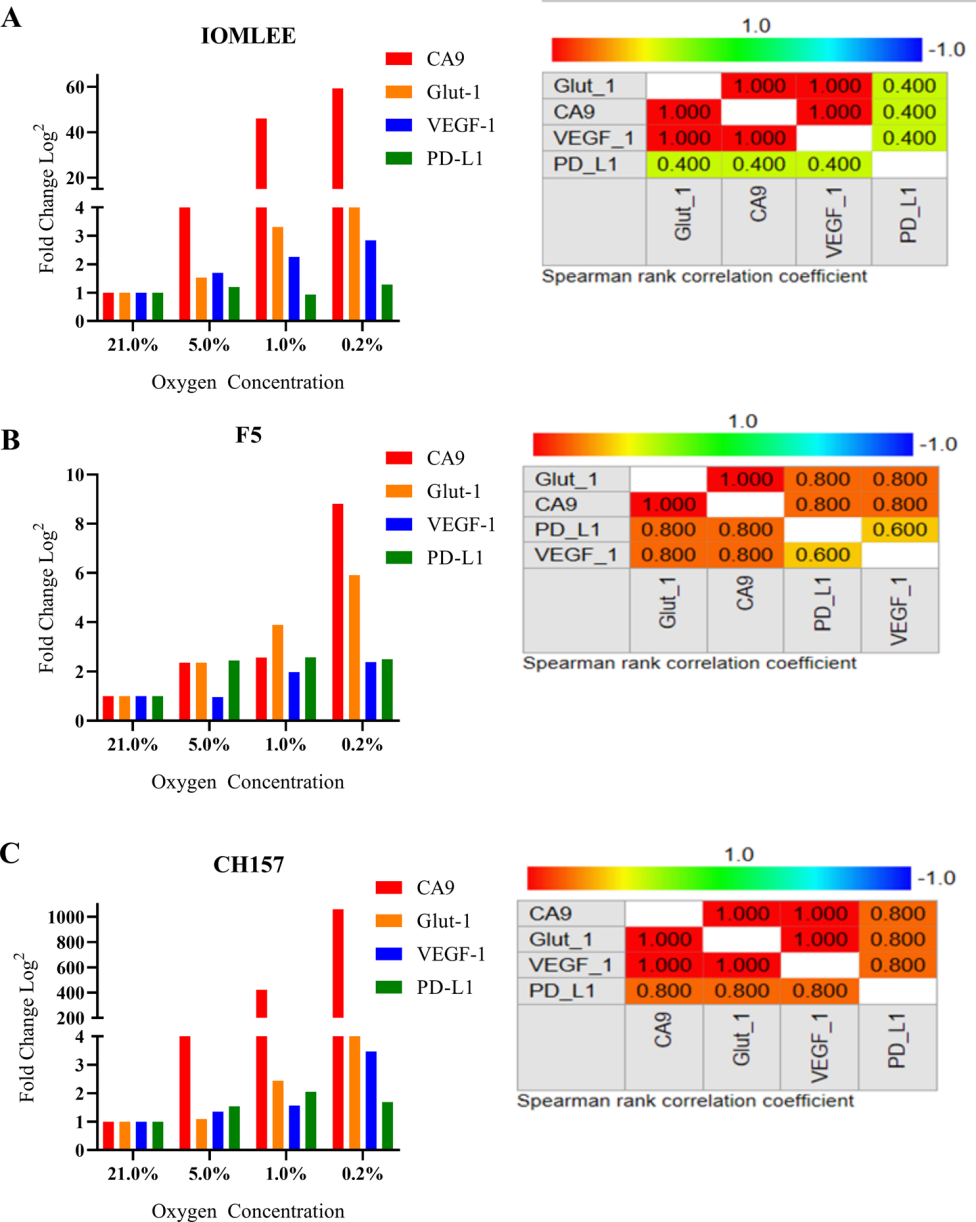
**A–C** The Bar graphs and spearman’s correlation demonstrate significant positive correlation of the mRNA expression (log2 transformation) of hypoxic markers (CA9, Glut-1 and VEGF-1) and PD-L1 in three meningioma cell lines; IOMLEE, F5 and CH157 under hypoxic conditions. (MedCalc).

In order to assess the positive effect of hypoxia on PD-L1 expression, we treated three malignant meningioma cell lines (F5, IOM-LEE, and CH157) with multiple low oxygen concentrations (0.2, 1, and 5%) and normoxia (21% O_2_, Control). The cells were harvested 48 h later and subjected to real-time PCR analysis of mRNA expression to assess the relative expression of PD-L1 and hypoxic markers, including *CA9*, *VEGF-A*, and *GLUT-1* under hypoxic and normoxic conditions. Our findings indicated that decreased oxygen concentrations resulted in hypoxia highlighted by elevation of mRNA expression of *CA9, Glut-1* and *VEGF-A* (Fig. 4A–C). Using Spearman correlation, we observed significantly positive correlation of mRNA expression of PD-L1 with these three hypoxic biomarkers under hypoxia in meningioma cell cultures (Supplemental Table 4, Fig. 4A–C). This finding is in line with our observation in the cohort tumor samples (above), showing correlation between expression of PD-L1 and the hypoxic marker; CA9, supporting the possibility of hypoxia as one of the potential regulatory mechanisms for PD-L1 expression in meningioma.

## Discussion

The role of immune suppression in progression of meningioma in comparison to glioblastoma and other cancers is under investigated. Recently, PD-L1 has attracted a substantial attention in the immune-oncology of meningioma. In this study, we used both visual and digital quantification of PD-L1 expression and demonstrated the independent predictive potential of PD-L1 as one of the most important immunosuppressive factors in cancer immunity^8^ and we investigated alterations in several cellular processes associated with its expression and regulation in meningioma. Prior reports showed PD-L1 expression mostly based on tissue microarray in meningiomas, particularly higher expression in anaplastic meningioma^16,17,25^. Here, we validated these results on whole sections of meningioma and provided details of distinct patchy, regional expression of this protein in meningiomas for routine diagnostics. . Furthermore, we demonstrated intra- and inter-tumoral heterogeneity in PD-L1 immuno-reactivity with low median percentage of positive cells of 0.1% in meningioma.

We noted significantly positive relationship between higher PD-L1 expression and grade. We demonstrated the independent prognostic significance of expression of this immunosuppressive biomarker to predict tumor recurrence. In addition we observed direct correlation of PD-L1 positivity with age and maximum tumor diameter. These findings support the potential of clinical utility of PD-L1-expression in meningioma patients to predict risk for tumor progression and in selection of refractory and high grade meningioma patients for immunotherapy.

Using transcriptomic data, we discovered the cellular network and processes associated with PD-L1 immune reactivity and found that expression of PD-L1 is associated with up-regulation in cellular genes and processes including NFKB2 and hypoxia.

NFkB2 gene encodes a member of the NFKB pathway genes, which is involved in inflammation, neoplastic progression, and immune reactivity in several cancers including meningioma^26^. We found a link between high PD-L1 and NFKB2 expressions in meningiomas specially in high grade tumors. Notably, co-expression of both NFKB2 and PD-L1 in the tumors predicted worse RFS. It has been shown that NFKB has a binding site in human PD-L1 (CD274) promoter and upon binding, it regulates PD-L1 transcription in human monocytes^27^. In addition, the regulatory role of NFKB signaling pathway in PD-L1 expression has already been described in several cancers including non-small lung carcinoma^11,27,28^.

We investigated the link between hypoxia and PD-L1 expression in meningioma. Interestingly, we detected a heterogeneous pattern of hypoxia in almost half of the meningioma cases; highlighted by membranous CA9 positivity. We found a similar pattern of peri-necrotic positivity in both CA9 and PD-L1 immunohistochemistry staining of the high grade meningioma cases in our cohort. Furthermore, analysis of the interplay between hypoxia and PD-L1 expression in meningioma revealed a positive relationship between CA9 (a well-established hypoxia marker) positivity and PD-L1 protein expression in primary meningioma. We also demonstrated the prognostic significance of CA9 expression alone in outcome of meningiomas and also when it was co-expressed with PD-L1 to predict the risk for tumor recurrence in our cohort. Hypoxia is a well-known trigger for PD-L1 expression and immune suppression in different types of cancers; it has been demonstrated that PD-L1 promoter contains a hypoxia response element (HRE), indicating the possible association between hypoxia and immune suppression in tumorigenesis^29^. Of note, hypoxia has been shown as one of the main regulatory mechanisms of PD-L1 gene promoter activation, not only in malignant tumors, but also in non-malignant neoplasms such as paraganglioma^30–33^ , similar to the results of our study in the meningioma cohort.

In addition, it has already been shown that expression of CA9 is characteristic of aggressive meningiomas^34^ and that a positive correlation between CA9 with microvascular proliferation predicts tumor recurrence, although its mechanism has not been explored^35,36^.

Induction of hypoxia in our meningioma cell lines was associated with PD-L1 expression, supporting the hypothesis of a potential link between hypoxia and immune suppression in high grade meningiomas. Although these findings shed light on regulatory role of hypoxia in meningioma, more detailed mechanistic studies are required to uncover the interplay between hypoxia and activation of PD-L1 in this tumor.

In summary, our findings describe the histopathologic and clinical features of PD-L1 expression in meningioma and suggest its clinical utility in management of clinically aggressive meningiomas specially prediction of tumor recurrence in routine diagnostics. Furthermore, our study hypothesized a link between NFKB2, hypoxia and PD-L1 expression in meningioma. These findings may provide a foundation for further mechanistic molecular investigations and study on role of PD-L1 expression in clinical management and immunotherapy of high grade meningioma.

## Materials and methods

### Patient characteristics

Retrospectively, we included meningioma patients who underwent craniotomy and minimum of follow up for 2 years and available formalin fixed paraffin embedded tissue (FFPE) in the archive of pathology department, Toronto Western Hospital, University Health Network between 1998 and 2016. The samples used in this study were obtained through the “University Health Network Research Ethics Board” approved study and samples are from institutional tumor biobank that obtains tumor samples through informed consent from all subjects and no subjects were under 18. All experimental protocols and methods were performed in accordance with institutional (University Health Network) regulations. The demographic, clinical and health care data were collected from the electronic patient records. To study the impact of extent of resection (EOR) on recurrence free survival (RFS), we divided our patients into gross total resection (GTR) and subtotal resection (STR) based on postoperative MRIs reports and neurosurgeon notes. We defined tumor recurrence based on growth of residual tumor according to postoperative neuroimaging and clinical assessment. The Hematoxylin and Eosin (H&E) stained slides were reviewed to confirm the diagnosis. Maximum tumor diameter (MTD) was defined as the largest single diameter recorded in the neuroimaging report.

### Immunohistochemistry staining

The H&E and immunohistochemistry staining slides of the tumoral surgical samples in this cohort were reviewed by SK and KA to confirm the diagnosis, WHO grading and visual scoring. The archival paraffin-embedded samples from selected tumor block were retrieved. IHC staining was performed on 5-µm sections. The primary antibodies included anti-PD-L1 (Sp142, Spring Bioscience), anti-RELB (D4, Santa Cruz), anti-NFKB2 (p100/p52, Cell signalling), pSTAT3 (Tyr705, Cell signalling) and Carbonic Anhydrase IX/CA9 (Rabbit poly clonal antibody, NB100, 417). The primary antibodies were incubated overnight at 1:100, 1:50, 1/800, 1:400 and 1/1,000 dilutions, respectively. The antigen retrieval buffer for PD-L1, RELB and pSTAT3 was Tris Ph = 9 and for NFKB2 and CA9 was citrate Ph = 6. We applied secondary antibody for one hour at room temperature, followed by processing with DAKO polymer-HRP system, DAB and finally counterstaining in Mayer’s Haematoxylin.

The immunohistochemical expression PD-L1 and CA9 were visually scored as positive and negative based on a definition of positive immune reactivity as at least identification of one focus of clear PD-L1 or CA9 membranous positivity in the tumor. We visually scored cytoplasmic NFKB2 immunoreactivity and nuclear positivity for both pSTAT3 and RELB in two-tiered subjective scoring system: low and high expression.

### Image analysis for PD-L1 IHC expression

In addition of visual scoring, image analysis was used for calculation of percentage of PD-L1 IHC positivity in the tumor. Quantification of positive membranous and cytoplasmic staining for PD-L1 was performed with the image analysis software HALO (HALO Technology Consulting, Ontario, Canada). SK and OS were trained to use HALO in PD-L1 IHC digital analysis and established algorithm to define positive cells based on detection of membranous immunoreactivity. Using this algorithm, the percentage of PD-L1 positivity was calculated by number of positive cells/total number of positive and negative cellsx100 in whole tumor section.

### Gene set enrichment analysis (GSEA)

To characterize molecular alterations associated with PD-L1 expression, we performed GSEA using gene expression data (Affymetrix GeneChip) from two GEO datasets: GSE16581, GSE9438^23,24^. PD-L1 (CD274) was used as phenotypic labels and GSEA was based on total number of 97 meningioma patients. For the two GEO datasets: GSE16581 and GSE9438, we downloaded Affymetrix. CEL files from GEO website and imported them into Partek Genomics Suit software (Partek, St. Louis, USA) based on quantile normalization, log2 transformation, and RMA (Robust MultiArray Average) background correction.

We computed differential expression to do pathway analysis between two groups based on expression with PD-L1. GSEA of the top differentially expressed genes in the PD-L1 high group identified several significantly enriched pathways. Analyzed gene sets included validated hallmark signatures. We evaluated enrichment in phenotype both in positive and negative correlation with PD-L1. We examined Pearson correlation between RFS and PD-L1 RNA expression. We did signaling pathway analysis using the Database for Annotation, Visualization and Integrated Discovery (DAVID) based on significantly differentially expressed genes with a twofold expression difference.

### Cell culture

Three invasive and malignant meningioma cell lines: F5^37^ and IOMM-Lee^38^ and CH157-MN^39^ were cultured in Dulbecco’s modified Eagle’s medium (DMEM) supplemented with 10% fetal bovine serum (FBS) (Gibco/Invitrogen, NY, USA) at 37 °C in a humidified incubator (CO2 water-jacketed incubator; Thermo Electron, Waltham, MA) under 5% CO2/95% air. For hypoxia treatment, cells were seeded in 10 cm culture plates at a density of 1 × 10^6^ cells per plate in triplicate and transferred to a Whitley Hypoxic station H35 (Don Withley Scientific). Cells were exposed to hypoxia for 48 h in a (0.2, 1, or 5%) oxygen concentration, 5% CO2, 94% N2 gas mixture, followed by RNA extraction. Each experiment was repeated 3 times.

### RNA extraction and quantitative RT-PCR analysis of gene expression

We used TRIzol Reagent (Invitrogen, Carlsbad, CA, USA) to extract total cell RNA according to manufacturer’s instructions. Approximately 1 µg RNA was subjected to reverse transcription using the QuantiTect Reverse Transcription Kit (Qiagen, Cat # 205313). Real-time PCR was then performed on cDNA products using gene specific primers and Fast SYBR Green Master Mix (Cat. No. 4385612; Life Technologies) to quantify expression level of specific genes using the Step One Plus Real-Time PCR System (Life Technologies).

We analyzed the expression of PD-L1 and its regulatory genes. The primer sequences are as follows: PD-L1: 5′-GGTGCCGACTACAAGCGAAT-3′ and; GAPDH: 5′-ATCTCTGCCCCCTCTGCTGA-3′ and 5′-GATGACCTTGCCCACAGCCT-3′ and Glut-1 VEGF for CA9 (5′-TAGCGAGTGGTTCTTCTGCG-3′ and 5′-AGGGCTGCTGGA AGGTAAAC-3′). GLUT1: 5′-TGTGCAACCCATGGCTAA-3′ and 5′-CCTGGTCTCATCTGGATTCT-3′. VEGFA: 5′- AAGGAGGAGGGCAGAATCAT-3′ and 5′-ATCTGCATGGTGATGTTGGA-3′.

All RT-PCR reactions were performed using the following conditions: 95 °C for 2 min; 95 °C for 5 s, annealing and extension at 60 °C for 20 s for a total of 40 cycles. The amount of mRNA in each sample was normalized to the amount of GAPDH mRNA and relative abundance of individual mRNAs was determined by − 2ΔΔCt method.

### Statistical analysis

Chi-Squared, t-test, one way or two ways ANOVA test, Pearson’s or Spearman’s tests were used to analyze for any significant associations between variables as indicated. A *p* < 0.05 was used as a threshold for statistical significance. Kaplan–Meier (KM) curves and Cox proportional hazard regression analysis were used for univariate and multivariate survival analyses. R v3.3.1 (https://www.r-project.org/) and MedCalc statistical software (Version 19.2.0, Ostend, Belgium, www.medcalc.org) were used for all statistical analyses.

## Supplementary information

Supplementary information.
