# Supplementary material for: Programmed death ligand-1 (PD-L1) expression in meningioma; prognostic significance and its association with hypoxia and NFKB2 expression
Source: Sci Rep. 2020 Aug 24;10:14115. doi: 10.1038/s41598-020-70514-z (PMC7445252; doi:10.1038/s41598-020-70514-z)
Supplement: Supplementary file 1 — Supplementary information. [file 41598_2020_70514_MOESM1_ESM.docx]

**Authors:** Shirin Karimi^1^, Sheila Mansouri^1^, Yasin Mamatjan^1^, Jeff Liu^1^, Farshad Nassiri^1^, Suganth Suppiah^1^, Olivia Singh^1^, Kenneth Aldape^1, 2^, Gelareh Zadeh^1^*

**Affiliation:**

1- Princess Margaret Cancer Center

MacFeeters-Hamilton Center for Neuro-Oncology Research

14-701, Toronto Medical Discovery Tower (TMDT)

101 College St,

Toronto, ON M5G 1L7

2- National Cancer Institute

Building 10, Room 2S235

Bethesda, MD 20892–1500

***Corresponding author:** Dr. Gelareh Zadeh

Princess Margaret Cancer Center

MacFeeters-Hamilton Center for Neuro-Oncology Research

14-701, Toronto Medical Discovery Tower (TMDT)

101 College St,

Toronto, ON M5G 1L7

Email: **Gelareh.Zadeh@uhn.ca**

**Supplemental Table 1. Co-expression of PD-L1 with NFKB2 and CA9 in the meningioma cohort**

| **Character** | **NFKB2 expression *** | | **CA9 expression**** | |
| --- | --- | --- | --- | --- |
|  | Low | High | Negative | Positive |
| **PD-L1 expression (%) median=0.08** |  |  |  |  |
| **Low** | 34 | 8 | 19 | 17 |
| **High** | 28 | 18 | 16 | 20 |
| **Total** | 70.5% (62/88) | 29.5% (26/88) | 48.5%(35/72) | 51.4% (37/72) |

**Supplemental Table 2. Multivariate analysis of PD-L1 coexpression with NFKB2 and CA9 expression**

|  | **p** | **HR** | **CI (95%)** |
| --- | --- | --- | --- |
| **Co-expression of PD-L1 and NFKB2** | 0.002 | 1.57 | 1.117 to 2.11 |
| **WHO grade** | 0.02 | 1.85 | 1.08 to 3.16 |
| **Extent of Resection** | 0.007 | 2.86 | 1.33 to 6.15 |
|  |  |  |  |
| **Co-expression of PD-L1 and CA9** | 0.009 | 1.7 | 1.14 to 2.55 |
| **WHO grade** | 0.11 | 1.61 | 0.89 to 2.92 |
| **Extent of Resection** | 0.02 | 2.48 | 1.09 to 5.64 |

**Supplemental Table 3.Multivariate analysis of co-expression of PD-L1, NFKB2 and CA9 in meningioma**

|  | **HR (95%CI)** | **P** | **P (ANOVA)** | **Reference** |
| --- | --- | --- | --- | --- |
| **HighPD-L1-HighNFKB2-Negative CA9** | 1 (0.1-14.3) | 0.995 | 0.024 | Low-Low-Negative |
| **High PD-L1-High NFKB2--Positive CA9** | 8 (1.8-35.7) | 0.007 | 0.024 | Low-Low-Negative |
| **High PD-L1-Low NFKB2-Negative CA9** | 0.8 (0.2-3.4) | 0.77 | 0.024 | Low-Low-Negative |
| **HighPD-L1-Low NFKB2-Positive CA9** | 4 (0.8-19) | 0.08 | 0.024 | Low-Low-Negative |
| **Low PD-L1-High NFKB2-Negative CA9** | 4.2 (0.7-25.6) | 0.12 | 0.024 | Low-Low-Negative |
| **Low PD-L1-High NFKB2-Positive CA9** | 1 (0.1-7.8) | 0.97 | 0.024 | Low-Low-Negative |
| **Low PD-L1-Low NFKB2-Positive CA9** | 1.8 (0.5-6.5) | 0.39 | 0.024 | Low-Low-Negative |
| **Extent of resection** | 2.8 (1.1-6.9) | 0.03 | 0.13 | 0 |
| **WHO_grade (II vs I)** | 1.9 (0.8-4.6) | 0.13 | 0.2 | I |
| **WHO_grade (III vs I)** | 3.9 (0.7-22.3) | 0.13 | 0.2 | I |

**Supplemental Table 4. mRNA expression of hypoxic markers and PD-L1 in 3 malignant meningioma cell lines**

|  |  |  | **Oxygen concentration (%)** | | | |
| --- | --- | --- | --- | --- | --- | --- |
| **Meningioma cell line** |  |  | **21** | **5** | **0.01** | **0.002** |
| **IOMLEE** | **RNA expression (log2 Fold change)** | **CA9** | 1 | 10.275 | 46.075 | 59.25 |
|  |  | **Glut-1** | 1 | 1.534633 | 3.312759 | 6.791252 |
|  |  | **VEGF-1** | 1 | 1.700938 | 2.255287 | 2.842665 |
|  |  | **PD-L1** | 1 | 1.195 | 0.93 | 1.29 |
| **F5** |  | **CA9** | 1 | 2.36 | 2.57 | 8.81 |
|  |  | **Glut-1** | 1 | 2.36024 | 3.892732 | 5.911555 |
|  |  | **VEGF-1** | 1 | 0.961339 | 1.98345 | 2.390289 |
|  |  | **PD-L1** | 1 | 2.445 | 2.575 | 2.5 |
| **CH157** |  | **CA9** | 1 | 50 | 422.5 | 1059.5 |
|  |  | **Glut-1** | 1 | 1.088247 | 2.441909 | 7.064172 |
|  |  | **VEGF-1** | 1 | 1.352127 | 1.572501 | 3.469632 |
|  |  | **PD-L1** | 1 | 1.54 | 2.045 | 1.685 |
